# Aging and Sequential Strategy Interference: A Magnetoencephalography Study in Arithmetic Problem Solving

**DOI:** 10.3389/fnagi.2018.00232

**Published:** 2018-08-08

**Authors:** Angélique Roquet, Thomas Hinault, Jean-Michel Badier, Patrick Lemaire

**Affiliations:** ^1^Aix-Marseille Université & CNRS, Marseille, France; ^2^Department of Psychological and Brain Sciences, Johns Hopkins University, Baltimore, MD, United States; ^3^Aix-Marseille Université, INS, Marseille, France; ^4^INSERM U1106, Marseille, France

**Keywords:** aging, strategy execution, arithmetic, cognitive control, magnetoencephalography

## Abstract

This study investigated age-related changes in the neural bases of sequential strategy interference. Sequential strategy interference refers to decreased strategy interference (i.e., poorer performance when the cued strategy is not the best) after executing a poorer strategy relative to after a better strategy. Young and older adults performed a computational estimation task (e.g., providing approximate products to two-digit multiplication problems, like 38 × 74) and were matched on behavioral sequential strategy interference effects. Analyses of magnetoencephalography (MEG) data revealed differences between young and older adults in brain activities underlying sequential strategy interference. More specifically, relative to young adults, older adults showed additional recruitments in frontal, temporal, and parietal regions. Also, age-related differences were found in the temporal dynamics of brain activations, with modulations occurring both earlier and later in older than young adults. These results suggest that highly functioning older adults rely on additional mechanisms to process sequential strategy interference as efficiently as young adults. Our findings inform mechanisms by which highly functioning older adults obtain as good performance as young adults, and suggest that these older adults may compensate deleterious effects of aging to efficiently execute arithmetic strategies.

## Introduction

The goal of the present study was to investigate age-related differences in the spatial-temporal dynamics of brain activations during sequential modulations of arithmetic strategy execution. We used magnetoencephalography (MEG) to document these brain activations while participants execute strategies to solve arithmetic problems. A strategy can be defined as “a procedure or a set of procedures for achieving a higher-level goal or task” (Lemaire and Reder, 1999, p. 365). Previous research on cognitive aging have found that young and older adults differ in the type and number of strategies they use to accomplish cognitive tasks, as well as how they select and execute available strategies. This has been found in a number of cognitive domains, including from pattern recognition, attention, memory, problem solving, decision making, and language and arithmetic processing (see Lemaire, 2016, for an overview). Of particular interest for the present study, are age-differences in how young and older participants execute strategies. The present study further investigates such differences be examining neural bases of strategy sequential interference which occur when participants execute strategies. It is based on previous findings showing that a subgroup of older adults is as able as young adults to process strategy interference. By examining age-related differences underlying strategy interference, the present study enables to understand how this occurs. The significance of these findings is important as they help us to further our understanding of how highly functioning older adults can compensate deleterious effects of aging to efficiently accomplish cognitive tasks in general, and to efficiently execute cognitive strategies in particular. Before outlining the logic of the present research, we first review previous findings on sequential modulations of strategy interference effects and discuss how important it is to examine such sequential modulations to further understand how young and older adults accomplish cognitive tasks.

Recent studies found that when participants execute strategies on a given trial to accomplish a cognitive task, they are influenced by which strategy was used on the immediately preceding trial. This has been found in several cognitive domains, like arithmetic (e.g., Uittenhove and Lemaire, 2013a,b; Hinault et al., 2014, 2016a, 2017; Lemaire and Hinault, 2014; Hinault and Lemaire, 2016) or episodic memory (e.g., Hinault et al., 2016a,b; Burger et al., 2017). As an example, Lemaire and Hinault (2014) found sequential modulations of strategy execution during the arithmetic problem solving tasks. They used a computational estimation task (i.e., participants had to estimate the product of multiplication problems, such as 46 × 72, without calculating the exact product) and asked participants to execute a specific rounding strategy on each problem. For each problem, the cued strategy could be the better strategy (i.e., the strategy that yields the closest estimate to the correct product) or the poorer strategy. The authors observed strategy interference effects (i.e., reduced performance when the cued strategy was the poorer strategy relative to the better strategy). Moreover, strategy interference effects on a given problem were modulated by the strategy executed on the immediately preceding problems. Indeed, strategy interference effects were smaller following the execution of the poorer strategy, relative to after executing the better strategy. These modulations suggest the implication of cognitive control processes to detect (and resolve) an interference (between the strategy activated upon problem encoding and the required strategy) on poorer strategy problems. This in turn led the cognitive system to increase its level of control in order to resolve strategy interference more efficiently on the next problem. That is, after detecting an interference on poorer strategy problems, participants increased their level of top-down control on current problems so as to increase processing of strategy-relevant information (e.g., cued strategy) on a given problem and decrease activation of the strategy irrelevant information (e.g., size of unit digits). Conversely, on better strategy problems, participants did not prepare themselves for subsequent poorer strategy problems and were less efficient at interference processing (see Diamond, 2013, for a review).

The goal of the present study was to investigate age-related differences in sequential modulations of arithmetic strategy execution. We used magnetoencephalography (MEG) to document the spatial-temporal dynamics of brain activations and aging effects therein.

Interestingly, Lemaire and Hinault (2014) found important individual differences in how sequential strategy interference effects evolve with age. Two sub-groups of older adults were distinguished based on independent measures of cognitive control processes. So-called “high-control” older adults showed similar sequential strategy interference effects than young adults, in contrast to “low-control” individuals (who showed reverse sequential modulations of strategy interference effects). Individual differences among older adults in sequential modulations of strategy interference effects reported by Lemaire and Hinault (2014) shed new lights on age-related differences in sequential modulations of executive control processes previously reported in conflict tasks (e.g., Simon, Stroop, flanker, and Go/No-go tasks). Indeed, whereas Monti et al. (2010) showed age-related declines in sequential adjustments of cognitive control processes from one trial to the next (e.g., Nessler et al., 2007; Lucci et al., 2013), several studies reported age-related invariance in sequential modulations (e.g., West and Moore, 2005; Puccioni and Vallesi, 2012; Joyce et al., 2014; Larson et al., 2016). One possible source of such contradictory findings, as suggested by Lemaire and Hinault (2014)'s study, concerns individual differences among older adults (e.g., Maddox and Douglas, 1974; Li et al., 2001; Hedden and Gabrieli, 2004; Goffaux et al., 2008). Depending on tested samples, individual differences during aging may lead to observe age-related changes in sequential modulations of cognitive control (i.e., if samples of older adults include mostly “low-control” individuals) or to find no age differences therein (i.e., if samples of older adults include mostly “high-control” older individuals or a mixed of low- and high-control individuals). As Lemaire and Hinault (2014)'s findings suggest, it is important to control for these differences to investigate age-related differences and similarities in sequential effects during execution of cognitive strategies.

One hypothesis on which the present work is based is that age invariance in behavioral performance could be related to age differences in brain activations. Previous electroencephalography (EEG) studies investigated age-related changes in the time course of brain activities in older adults with similar sequential strategy interference effects relative to young adults (El Yagoubi et al., 2005; Goffaux et al., 2008; Hinault et al., 2016a; Larson et al., 2016). These common results revealed that similar behavioral sequential modulations in “high-control” older adults and in young adults were associated with earlier and later time windows of brain activations in older adults. Goffaux et al. (2008) revealed that young and older adults with high working memory had comparable behavioral performance in task-switching, in contrast to older adults with lower working memory. Both young and high performing older adults showed larger posterior negative slow-wave activities. However, high performing older adults also showed frontally distributed activities, which suggest that the recruitment of additional brain networks was associated with the maintenance of cognitive functioning.

Studies using functional magnetic resonance imaging (i.e., fMRI) investigated what characterizes older adults with similar behavioral performance than young adults (e.g., Grady et al., 1994; Reuter-Lorenz et al., 2000; Cabeza, 2002; Cabeza et al., 2002; Logan et al., 2002; Madden, 2007; Gandini et al., 2008; Angel et al., 2015). The common results also found that age invariance in behavioral performance was associated with age differences in neural activations. Using a recognition memory task, Angel et al. (2015) showed additional activations of the frontal gyrus in a so-called “Old high group” (i.e., older adults with high level of executive functioning), relative to the so-called “Old low group” (i.e., older adults with low executive performance). Indeed, results revealed larger bilateral activations in frontal gyrus in the “Old high group” compared to the “Old low group”. Theoretically, this was consistent with the Hemispheric Asymmetry Reduction in OLDer adults model (i.e., HAROLD model; Cabeza, 2002), according to which bilateral frontal activations are associated with a functional gain in “high-performing” older adults (e.g., Rypma and D'Esposito, 2000; Logan et al., 2002). However, given the low spatial (EEG) or temporal (fMRI) resolution of these brain imaging techniques, the spatial-temporal dynamics of additional brain activities in older adults are still poorly identified. Specifying how the time course of activations of brain regions differ between young and high performing older adults is critical to better understand the differences in cognitive processes engaged by these two age groups. Furthermore, in the majority of previous neuroimaging studies of age-related changes in the neural bases of cognitive performance, the type of strategy used by young and older adults was not controlled. Therefore, whether the observed differences in brain activations reflect changes in strategy execution or strategy repertoire is undetermined. In the present study, we aimed at further understanding how highly functioning older adults can compensate deleterious effects of aging to efficiently accomplish cognitive tasks in general, and to efficiently execute arithmetic strategies in particular.

Brain activations underlying sequential strategy interference effects were recently documented in a magnetoencephalography (MEG) study in young adults (Hinault et al., 2017). Results revealed both spatial and temporal dynamics of brain activations underlying sequential strategy interference effects in young adults. More specifically, activations of the Anterior Cingulate Cortex (i.e., ACC) were observed during the execution of the poorer strategy, following the execution of the better strategy. The ACC was previously described as associated with conflict detection and resolution when no preparation to process conflict has been engaged (e.g., Carter et al., 2000; Kerns et al., 2004; Braver et al., 2009). Conversely, when the previous problems were solved with the poorer strategy, Hinault et al. (2017) found activations of the Left Inferior Frontal Junction (i.e., LIFJ), before the onset of the next problems. The LIFJ was previously associated with the coordination of activations toward a goal and the preparation for conflict processing (e.g., Brass and Von Cramon, 2004; Brass et al., 2005; Montojo and Courtney, 2008; Braver et al., 2009). Unknown is whether these brain areas are also activated in highly performing older adults and, if that is the case, whether the time course of these brain activations is similar or different in young and older adults. This issue is addressed in the present study.

We matched young and older adults on behavioral sequential strategy interference effects to investigate differences in brain activations between young and “high-control” older adults. Based on previous findings in neurosciences of aging, we tested two sets of predictions. First, in line with Hinault et al. (2017), older adults were expected to activate similar brain areas than young adults (i.e., ACC and LIFJ) but with differences in magnitudes and durations (i.e., earlier and additional times windows). Second, we expected additional brain activations in frontal, parietal, and temporal regions in older adults (e.g., Gandini et al., 2008; Reuter-Lorenz and Park, 2014). More specifically, we predicted that older adults recruit additional regions associated with inhibitory and working memory processes (e.g., Niendam et al., 2012; Nee et al., 2013) both before onset of problems to improve proactive adjustments and during strategy execution to maintain highly efficient strategy execution. Such differences in brain activations were expected to be larger after the execution of a poorer strategy on the previous problem than following execution of the best strategy.

## Methods

### Participants

Fourteen older adults (eight men; 65–83 years of age; mean age: 72.5 years) participated in this experiment (see Table 1). To control for general cognitive abilities, we ensured that all older adults had a Mini Mental-State Examination (i.e., MMSE; Folstein et al., 1975) score of 27 or higher. No participant had a history of major medical problems, medications, major psychiatric illness, major head injury, or neurological disease. In order to study high-control older adults, we matched older participants with young adults (four men; 18–29 years of age; mean age: 22.1 years) tested by Hinault et al. (2017) on behavioral sequential strategy interference effects. Both groups showed similar behavioral sequential strategy interference effects (i.e., significant sequential strategy interference effects, with no interaction involving the age factor). The sample size used in this experiment was based on an a-priori power analysis conducted in G^*^Power 3.1 (Faul et al., 2007). Assuming an effect size of Cohen's *f (V)* = 0.75 (derived from relevant previously published studies; e.g., Hinault et al., 2016a, 2017), an alpha of 0.05, and two groups, we determined that a total sample size of 22 participants (*N* = 11 per group) would provide 83% power to detect effects. In order to exceed this criterion and achieve larger than 83% power, we recruited 28 participants (*N* = 14 per group). This study was approved by the local ethics committee, and a written informed consent was obtained from each participant prior to the experiment. All participants were paid 20 Euros for their participation. This study was carried out in accordance with the recommendations of the local ethic Committee (Comité de Protection des Personnes Sud-Méditerranée II, Ref: 217 R32; agreement N°2016-A00R23-48). All subjects gave written informed consent in accordance with the Declaration of Helsinki.

**Table 1:** Participants' characteristics.

**Variables**	**Young adults (*N* = 14)**	**Older adults (*N* = 14)**	***df***	***MSe***	***F***	***p***
Age in years and months	22.1	72.5	–	–	–	–
Years of education	15.3	14.9	1,27	1.29	0.14	0.712
MHVS	23.9	27.9	1,27	112.00	10.12	0.004
Arithmetic fluency	54.8	71.5	1,27	1955.57	3.50	0.073
MMSE	–	29.2	–	–	–	–

*MHVS, French version of the Mill-Hill Vocabulary Scale (Raven, 1951; Deltour, 1993). MHVS consists of 33 items distributed across three pages. Each item was a target word followed by six proposed words, and the task consisted in identifying which word was the closest to the target. Arithmetic fluency, Score obtained in a paper-and-pencil arithmetic test (French Kit; French et al., 1963) in which participants have to solve as many basic arithmetic problems (e.g., addition, subtraction, and multiplication problems as possible in 8 min; MMSE, Mini Mental State Examination; Folstein et al., 1975). None of the older adults obtained MMSE score lower than 27; therefore, none were excluding. Participants took the MHVS and then the arithmetic fluency tests after the computational estimation task*.

### Stimuli

Participants were asked to estimate products of multiplication problems. Each of the 208 sequences was made of two consecutives two-digit multiplication problems (e.g., 46 × 72). Following previous findings in arithmetic (see Cohen Kadosh and Dowker, 2015, for a review), we controlled the following factors: (a) no operand had a zero unit digit, (b) no operand had five as unit digit, (c) no digits were repeated within operands, (d) no reverse orders of operands were used, (e) the first operand was larger than the second in half the problems, and vice versa in the other problems, (f) no operand had its closest decade equal to 0, 10, or 100, (g) differences between correct products and estimates (i.e., result of the estimation strategy) were matched across strategies (i.e., mean percent deviations were identical between the mixed-rounding up-down and the mixed-rounding down-up strategy on all problems), and (h) rounded operands were never the same across the two problems of a given sequence.

Half the problems were mixed-rounding up-down problems, and half were mixed-rounding down-up problems. The unit digit of the first operand was smaller than five, and that of the second operand was larger than five in the mixed-rounding down-up problems (e.g., 54 × 36). It was the reverse in the mixed-rounding up-down problems (e.g., 46 × 72). Two types of problems were tested: Better strategy and poorer strategy problems. On better strategy problems, the cued strategy matched the problem type: Mixed-rounding down-up problems were cued with the mixed-rounding down-up strategy (e.g., doing 50 × 40 to estimate 54 × 36), and mixed-rounding up-down problems were cued with the mixed-rounding up-down strategy (e.g., doing 50 × 70 to estimate 46 × 72). Conversely, the cued strategy and the problem type differed on poorer strategy problems. Poorer strategy and better strategy problems were matched on correct products and on mean percent deviations between correct products and estimates.

Four types of sequence were tested (see examples in Table 2): Better-better sequences (i.e., both current and previous problems were solved with the better strategy), better-poorer sequences (i.e., current problems were solved with the poorer strategy and previous problems with the better strategy), poorer-better sequences (i.e., current problems were solved with the better strategy and previous problems with the poorer strategy), and poorer-poorer sequences (i.e., both current and previous problems were solved with the poorer strategy). We focused on “better-poorer” and “poorer-poorer” sequences to test how older adults sequentially modulated strategy interference on a given problem as a function of the interference on the immediately preceding problems (e.g., Uittenhove and Lemaire, 2012, 2013b; Lemaire and Hinault, 2014; Hinault et al., 2016a, 2017). The rationale for contrasting these two sequences was that poorer–poorer and better–poorer sequences were expected to reveal distinct neuro-physiological patterns on current poorer strategy problems, as a function of the strategy executed on previous problems. Strategy repetition and alternation were controlled. The cued strategy was identical for both problems in half the sequences, and different in the other sequences. This resulted in equal proportions of switch and no-switch problems in each condition. This design was used to prevent any alternative interpretation of sequential effects in terms of cue-switch costs.

**Table 2:** Four types of sequences tested in this study.

	**Previous problems: Better strategy**	**Previous problems: Poorer strategy**
Current problems: Better strategy	**Better–Better** *23 × 47 (DU) – 68 × 12 (UD)*	**Poorer–Better** *23 × 47 (UD) – 68 × 12 (UD)*
Current problems: Poorer strategy	**Better–Poorer** *23 × 47 (DU) – 68 × 12 (DU)*	**Poorer–Poorer** *27 × 43 (DU) – 68 × 12 (DU)*

*Sequences are defined by the cued strategy on the previous problems (better, poorer) and on the current problems (better, poorer). The letters indicate whether the rounding down-up strategy (DU) or the rounding up-down strategy (UD) was cued*.

### Procedure

**Figure 1:**
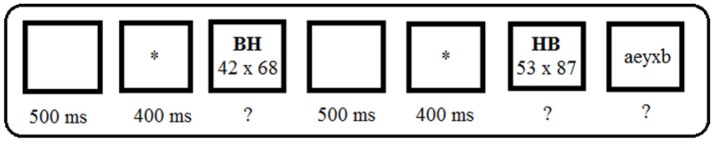
Events within a sequence. The letters “BH” (i.e., standing for “down-up” in French) cued participants to use the mixed-rounding down-up strategy, and the letters “HB” (i.e., standing for “up-down” in French) prompted participants to use the mixed-rounding up-down strategy.

The experiment was implemented using the E-Prime software (Psychology Software Tools, 1999). Each sequence began with a blank screen of 500 ms, followed by a warning signal (“^*^”) presented for 400 ms in the center of the screen (see Figure 1).

The problem and the cue were then simultaneously displayed on the computer screen. The cue appeared 2 cm above the problem. Both the problem and the cue remained on the screen until participants' response. The letter string “BH” (standing for “Down-Up” in French) cued participants to use the mixed-rounding down-up strategy, while “HB” (standing for “Up-Down” in French) cued them to use the mixed-rounding up-down strategy. Participants provided their response aloud. To reduce MEG signal contamination by speech articulation, and following previous studies (Lemaire et al., 2004; Ardiale and Lemaire, 2012; Uittenhove and Lemaire, 2013b; Lemaire and Hinault, 2014), participants were asked to vocalize only their final answer, after which the next problem was manually triggered by the experimenter. Errors in strategy selection were defined as participants not using the cued strategy. Errors in strategy execution were defined as participants failing to correctly execute the procedures of the cued strategy. Following the participants' oral response, a blank screen was presented for 500 ms, followed by a “^*^” warning signal for 400 ms. The second problem of a sequence was then presented, together with the corresponding cue. Then, after a 500-ms blank screen, “^*^” appeared for 400 ms, followed by a five-letter string (e.g., *aeiou*). Participants had to press the “L” key on an AZERTY keyboard if all letters were either vowels or consonants, or the “S” key if letters included both vowels and consonants (i.e., half the five-letter strings included either consonants or vowels only, and half included both types of letters). Following previous works (e.g., Ardiale et al., 2012; Lemaire and Hinault, 2014), this letter-judgement task avoids interference between the last problem of a sequence and the first problem of the next sequence. A 1,000-ms blank screen was displayed before the next sequence started.

The MEG experiment consisted of four blocks of 52 sequences each, with 5-min breaks between blocks. The order of presentation was counterbalanced across participants. Each session lasted about 45–60 min. Participants were instructed to estimate the product of multiplication problems as fast and accurately as possible using only the cued strategy. The two mixed-rounding strategies were then explained to participants. The mixed-rounding down-up strategy was described as rounding the first operand down to the nearest decade and the second operand up to the nearest decade, for instance doing 40 × 70 to estimate 43 × 68. The mixed-rounding up-down strategy was described as rounding the first operand up to the nearest decade and the second operand down to nearest decade, for instance, doing 40 × 60 to estimate 38 × 64. The participants started with a practice phase included eight sequences (each involving two multiplication problems, and a series of five letters).

### MEG recording

The data were acquired at La Timone Hospital in Marseille, using a 248-channel whole-head 4D Neuroimaging MEG system, at a sampling rate of 2,035 Hz. The electrooculogram and the electrocardiogram were recorded to capture eye movements and heartbeats, respectively. Five head-positioning coils were attached to the forehead and to the periauricular points to determine the position of the head. The individual head shape, consisting of the forehead, nose, and the location of the head-position coils were digitized (Polhemus Fastrak, Polhemus Inc., Colchester, VT, USA). Participants were lying on a hospital bed inside a magnetically shielded room. Stimuli were presented on a 800 × 600 resolution screen placed about 45 cm above participants, using a 48-point bold courier font (black color), using a standard video projector. The visual angle was 1.4°. Head position inside the MEG helmet was measured at the beginning of every block. Head displacements were monitored for remaining under 5 mm within each block. The exact timing of visual presentation was captured using photo-diodes that detected brightness changes on the presentation screen.

### MEG analyses

Artifact and channel rejection (on continuous data), filtering (0.1–20 Hz bandpass, on unepoched data), time segmentation into 12.40-s epochs, averages, and source estimation were all performed using Brainstorm (Tadel et al., 2011). Continuous data were visually inspected to identify physiological (e.g., blinks, saccades, heartbeats) and non-physiological (e.g., bad sensors) artifacts. Epoching of problem was time-locked to the onset of the first problem, and included 400-ms of pre-stimulus baseline. We selected this period as baseline, as the period before the second problem of a sequence was assumed to be influenced by the processing of the first problem. Artifact free epochs were extracted from −400 to 1,500 ms around the second problem of a sequence. Artifact-free epochs for each experimental condition were averaged separately to obtain event-related magnetic fields (ERFs) in each participant. The average numbers of epochs (±standard deviations) in analyses for poorer-better sequences, and poorer-poorer sequences were, respectively of 46 (±6) problems, and 44 (±7) problems in young adults, and 48 (±6) problems, and 49 (±6) problems in older adults, with a minimum number of 35 problems for one participant in one condition.

A free-orientation, cortically constrained minimum-norm estimation (MNE) procedure was applied to estimate the cortical origin of the brain responses (Hämäläinen and Ilmoniemi, 1994; Hauk, 2004). The MNE was weighted by a sample estimate of sensor noise covariance matrix (Dale et al., 2000) obtained from 30 s of empty room recording, in each of the participants, and used for improved data modeling, as typical in MNE approaches (e.g., Baillet et al., 2001). The MEG forward model was obtained from overlapping spheres fitted to each participant's scalp points (Huang et al., 1999). For all participants, sources were constrained to a cortical surface mesh template obtained from the MNI (i.e., Montreal Neurological Institute) Colin 27 brain. Brainstorm was used with default parameters to deform the template to each participant's digitized head shape (see Leahy et al., 1998, for technical details). The norm of the three source time series at each cortical voxel (i.e., conversion of orientation-unconstrained sources to flat maps, taking the norm of the three elementary dipoles at each time step, yielding only one value by vertex) was extracted and *z*-scored with respect to the pre-stimulus ([−400, 0] ms) baseline.

Following Hinault et al.'s results (Braver et al., 2009; Hinault et al., 2017), we expected to observe activations of ACC and the LIFJ. Moreover, we were interested to determine whether brain activations could be observed elsewhere in the scalp to determine all brain areas activated in “highly functioning” older adults. A whole-brain *permutation*-test (*p* < 0.001, uncorrected) was run to determine whether significant activations were present in other brain areas than the young adults' Region Of Interest (i.e., ROIs). Following previous neuroimaging studies on sequential modulations of cognitive control processes (e.g., Kerns et al., 2004; Hinault and Lemaire, 2016; Hinault et al., 2017), we compared better-poorer sequences (i.e., the current problems were solved with the poorer strategy after the previous problems were solved with the better strategy) to poorer-poorer sequences (both current and previous problems were solved with the poorer strategy). The rationale for contrasting these two sequences was that poorer-poorer sequences and better-poorer sequences were expected to reveal distinct neurophysiological patterns on current poorer strategy problems as a function of the strategy executed on previous problems. To assess when brain areas are significant and how brain activations differ between ROIs, we then used a 2 (Strategy on the previous problems: Poorer, better) × 15 (ROIs) × 36 (Times) repeated design ANOVA, with FDR and Sidak corrections to control for Type 1 error (e.g., Benjamini and Hochberg, 1995; Abdi, 2007). We averaged brain activations over periods of 50 ms (−400 to 1,500 ms after presentation of the problems) in the selected ROIs (defined using the Desikan Atlas; Desikan et al., 2006; ROIs resumed in Table 3).

**Table 3:** Coordinates of the brain activations over periods of 50 ms (−400 to 1,500 ms after presentation of the problems), defined using the Desikan Atlas (Desikan et al., 2006).

**ROIs**	**Abbreviations**	**Coordinates of the center**			**Width on the cortex (cm^2^)**
		***x***	***y***	***z***	
Left anterior cingulate cortex	ACC	98	164	89	11.71
Left inferior frontal junctions	LIFJ	36	141	107	9.13
Right inferior frontal junctions	RIFJ	148	147	102	22.00
Left prefrontal cortex	LPFC	87	207	68	11.10
Right prefrontal cortex	RPFC	112	203	59	14.50
Left precentral gyrus	LPreCG	53	126	135	7.52
Left orbitofrontal cortex	LOFC	58	178	73	5.93
Left median frontal gyrus	LMFG	74	178	97	8.94
Right median frontal gyrus	RMFG	137	174	100	7.40
Left superior frontal gyrus	LSFG	122	170	107	5.01
Right superior frontal gyrus	RSFG	79	202	88	4.63
Left superior temporal gyrus	LSTG	144	106	74	12.42
Right superior temporal gyrus	RSTG	35	129	78	4.18
Left inferior parietal lobule	LIPL	34	97	115	7.70
Right inferior parietal lobule	RIPL	162	116	94	3.36

Additional statistics were performed on ROIs to assess the significance of changes in activation between ROIs and over time. Unless otherwise noted, only effects significant to at least *p* < 0.05 were reported.

## Results

Results are reported in two main parts. First, we examined age-related differences in participants' performance (i.e., accuracy and response times). Then, we investigated age-related differences in the spatial-temporal dynamics of brain activations during sequential modulations of arithmetic strategy execution. In all the results, unless otherwise noted, differences are significant to at least *p* < 0.05.

### Behavioral results

Mean estimation times and error rates in strategy execution on the second problems of a sequence were analyzed using 2 (Group: Young adults, older adults) × 2 (Gender: Men, Women) × 2 (Strategy on the previous problems: Poorer, better) × 2 (Strategy on the current problems: Poorer, better) mixed-design ANOVAs, with age as the only between-participants factor. Preliminary analyses included verbal (e.g., Mill Hill) and arithmetic fluency (e.g., French kit) scores as co-variables. No main or interaction effects involving these factors came out significant, ruling out alternative explanations in terms of differences in arithmetic and/or verbal skills. No significant interaction was observed with genders (*F* < 1.0).

**Figure 2:**
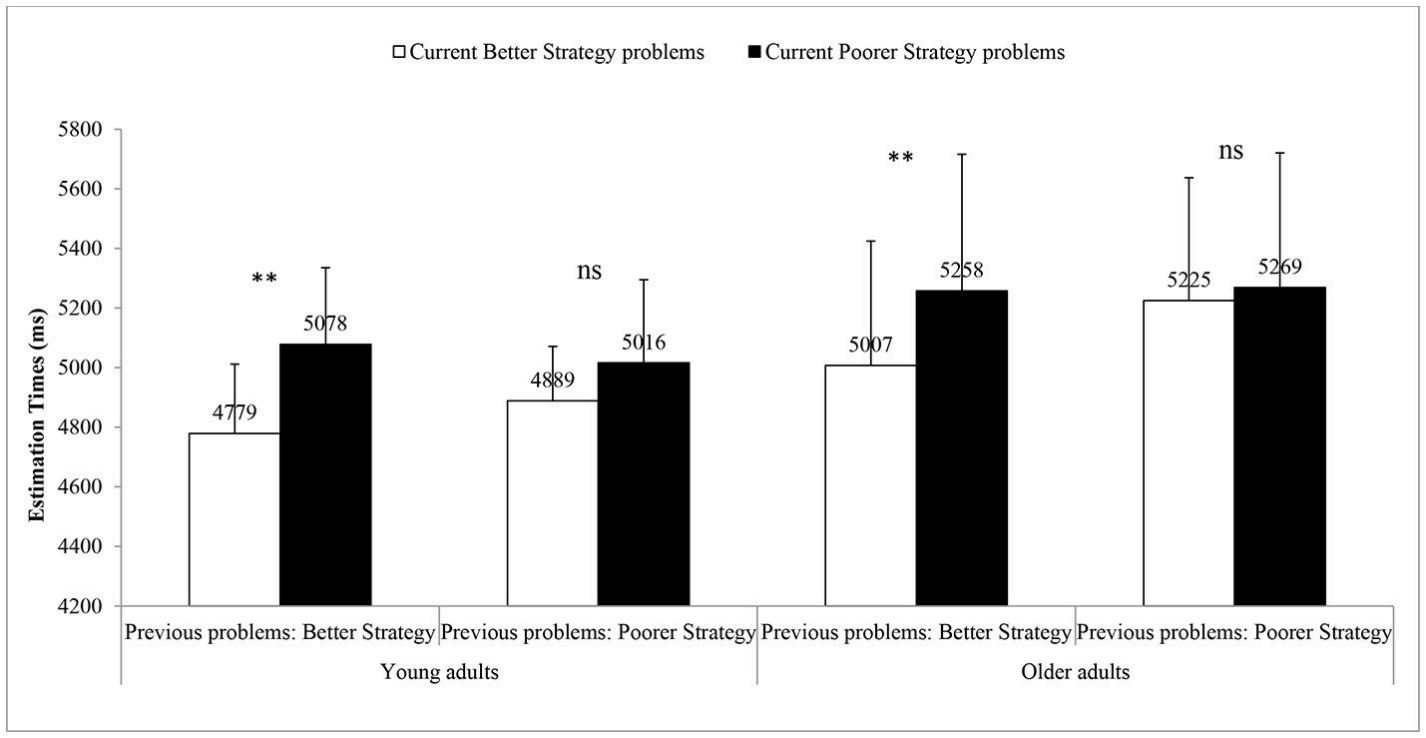
Mean solution times for current better strategy and poorer strategy problems following better strategy or poorer strategy problems in young and older adults. Error bars represent S.E.M. **p < .01, ns, non-significant.

The main effect of strategy on the current problems revealed that participants were slower on current poorer strategy problems than on current better strategy problems [i.e., 5,156 vs. 4,975 ms; *F*_(1, 26)_ = 13.34, *Mean Square error (MS*e) = 911,391, *partial eta squared* (η^2^_*p*_) = 0.34, *p* = 0.001; see Figure 2]. Also, the Strategy on the previous problems x Strategy on the current problems interaction was significant [*F*_(1, 26)_ = 14.56, *Ms*e = 250,366, η^2^_*p*_ = 0.36, *p* = 0.001]. Most importantly, the Group x Strategy on the previous problems x Strategy on the current problems was not significant (*F* < 1.0). This showed that sequential strategy interference effects (i.e., on better-poorer and poorer-poorer sequences) were of comparable magnitudes in both young adults [i.e., 172 ms; *F*_(1, 13)_ = 5.88, *MS*e = 104,093, η^2^_*p*_ = 0.31, *p* = 0.031] and older adults [i.e., 206 ms; *F*_(1, 13)_ = 14.56, *MS*e = 148217, η^2^_*p*_ = 0.41, *p* = 0.011]. Similarly, we found comparable magnitudes of sequential strategy effects in both young and older adults in other sequences (i.e., better-better, poorer-better; *F* < 1.0).

Analyses of errors in strategy execution revealed only a significant main effect of age, [*F*_(1, 26)_ = 5.11, *Ms*e = 166, η^2^_*p*_ = 0.16, *p* = 0.032], as older adults made more errors than young adults (4.5 vs. 2.1%, respectively). Analyses of errors in strategy selection revealed that participants made fewer errors on current better strategy problems (i.e., 2.8%) than on current poorer strategy problems [i.e., 4.9%, *F*_(1, 26)_ = 9.36, *MS*e = 123, η^2^_*p*_ = 0.27]. Also, the significant interaction between strategy on the previous problems and strategy on the current problems [*F*_(1, 26)_ = 7.86, *MS*e = 56, η^2^_*p*_ = 0.23] revealed larger strategy interference effects on current problems following better strategy problems [i.e., 3.5%; *F*_(1, 26)_ = 19.79, η^2^_*p*_ = 0.43] than after poorer strategy problems (i.e., 0.7%; *F* < 1.0). Most importantly, the Age × Strategy on the previous problems × Strategy on the current problems interaction was also significant [*F*_(1, 26)_ = 5.71, *MS*e = 40, η^2^_*p*_ = 0.18]. Differences in strategy interference effects after better and after poorer strategy problems were smaller in young adults than in older adults (i.e., 0.14 vs. 5.22%). This result showed that strategy sequential interference effects were smaller in young adults than in older adults.

### MEG results

#### Brain activations in high functioning older adults

**Figure 3:**
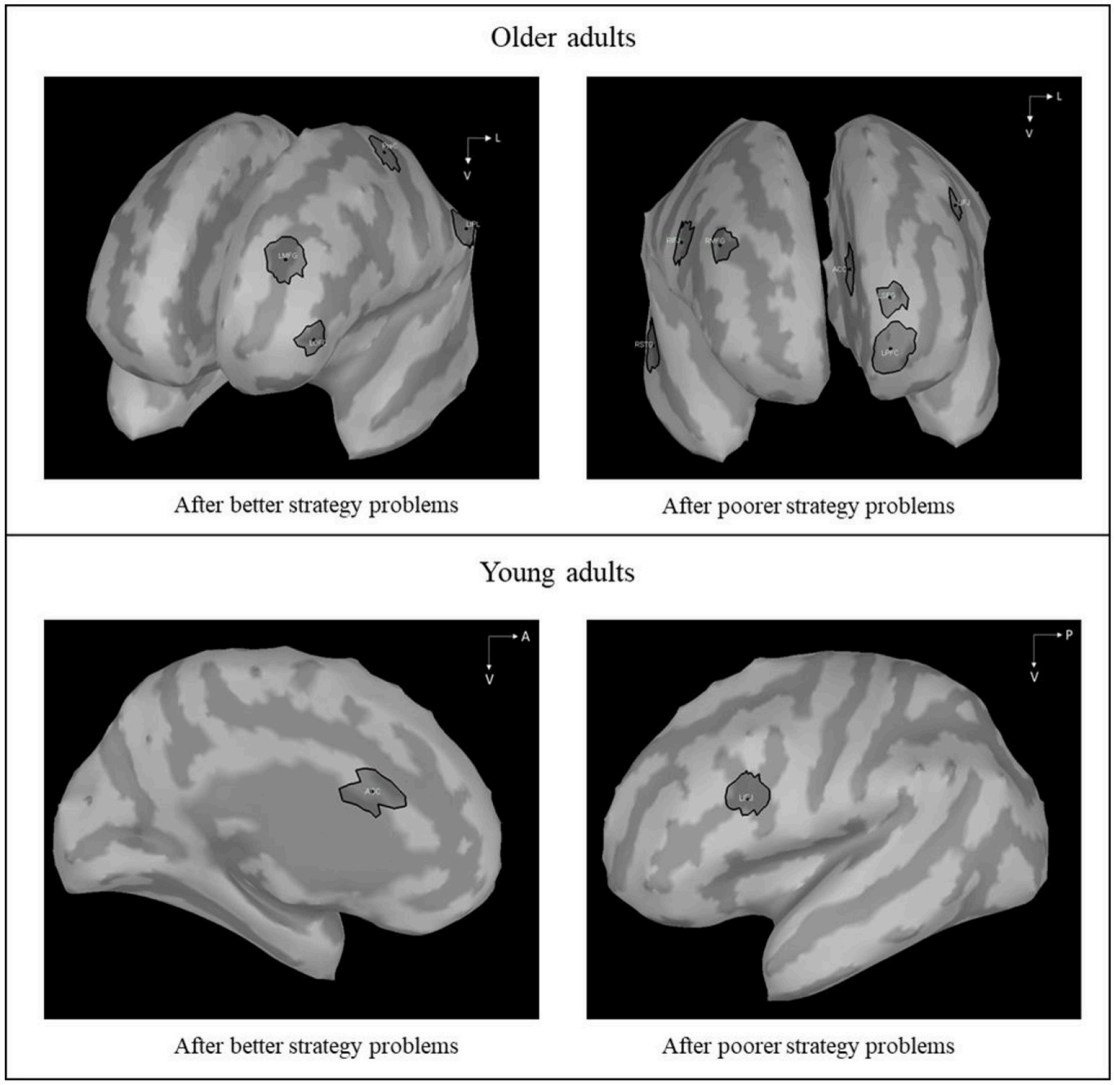
Differences in z-scored amplitudes, on current poorer strategy problems, between previous better strategy problems and following previous poorer strategy problems in older adults comparatively in young adults (Hinault et al., 2017). In older adults, when the second problems were preceded by the execution of the poorer strategy, significant brain activations were found in ACC (Anterior Cingulate Cortex), LIFJ (Left Inferior Frontal Junction), RIFJ (Right Inferior Frontal Junction), RPFC (Right Prefrontal Cortex), LMFG (Left Median Frontal Gyrus), RSFG (Right Superior Frontal Gyrus), LSFG (Left Superior Frontal Gyrus), and RSTG (Right Superior Temporal Gyrus) between −400 and −200 ms before the second problems display and between 500 and 650 ms following the onset of the second problems. When the second problems were preceded by the execution of the better strategy, activations were found in LMFG (Left Middle Frontal Gyrus), LPreCG (Left Precentral Gyrus), LOFC (Left Orbitofrontal Cortex), and LIPL (Left Inferior Parietal Lobule) between 100 and 350 ms and between 850 and 1,250 ms.

First, we were interested to determine the brain activations of “highly functioning” older adults on current poorer strategy problems as a function of whether previous problems were solved with the better or the poorer strategy. Following a whole-brain permutation test to identify the main activated ROIs, we conducted a 2 (Strategy on the previous problems: Better, poorer) × 15 (ROIs) × 38 (Time: Mean of 50-ms time windows) repeated measure ANOVA, with FDR and Sidak correction for multiple comparisons. The strategy on the previous problems × ROIs × Times interaction was significant [*F*_(555, 7, 215)_ = 1.28, *MS*e = 24.28, η^2^_*p*_ = 0.09, *p* = 0.09]. Contrasts revealed that different brain areas were activated after execution of the better strategy and after execution of the poorer strategy (see Figure 3), with distinct time courses for additional brain activations in older adults (i.e., frontal, temporal, and parietal regions; see Tables 4, 5). More specifically, larger amplitudes of brain activations for poorer-poorer sequences relative to better-poorer sequences were found in two time windows, between −400 and −200 ms before the second problems display and between 500 and 650 ms following the onset of the second problems, in frontal, and temporal regions (i.e., right median frontal gyrus). Conversely, larger amplitudes of brain activations for better-poorer sequences relative to poorer-poorer sequences were found between 100 and 350 ms and between 850 and 1,250 ms relative to the onset of the second problems, in frontal and parietal regions (i.e., left precentral cortex and left inferior parietal lobule). Furthermore, positive correlations were found between activations of ACC with frontal regions (see Table 6).

**Table 4:** Spatial-temporal dynamics of ROIs activated on current poorer strategy problems following better strategy problems, with zero being display of the second problems in older adults.

**ROI/TIME(ms)**	**0–100**	**100–150**	**150–200**	**200–250**	**250–300**	**300–350**	**350–850**	**850–900**	**900–950**	**950–1,100**	**1,100–1,150**	**1,150–1,200**	**1,200–1,250**	**1,250–1,500**
LPreCG			**FDR**	**FDR**										
LOFC														
LMFG														
RSFG														
LIPL				**FDR**										

*The colored boxes represent significantly larger activations in brain regions on current poorer strategy problems after better strategy problems than after poorer strategy problems. “FDR” showed brain areas still significant after FDR corrections, otherwise results were Sidak corrected. LPreCG, Left Precentral Gyrus; LOFC, Left Orbitofrontal Cortex; LMFG, Left Median Frontal Gyrus; RSFG, Right Superior Frontal Gyrus; LIPL, Left Inferior Parietal Lobule*.

**Table 5:** Spatial-temporal dynamics of brain areas activated on current poorer strategy problems following poorer strategy problems, with zero being display of the second problems in older adults.

**ROI/TIME (ms)**	**−400 to 350**	**−350 to 300**	**−300 to 250**	**– 250 to 200**	**−200 to 150**	**150–0**	**0–500**	**500–550**	**550–600**	**600–650**	**650–700**	**700–1,500**
ACC												
LIFJ												
RIFJ												
LPreCG												
LPFC												
LMFG												
RMFG		**FDR**										
RSFG												
LSFG												
RSTG												

*The colored boxes represent significantly larger activations in brain regions on current poorer strategy problems after poorer strategy problems than after better strategy problems. “FDR” showed brain areas still significant after FDR corrections, otherwise results were Sidak corrected. ACC, Anterior Cingulate Cortex; LIFJ, Left Inferior Frontal Junction; RIFJ, Right Inferior Frontal Junction; LPFC, Left Prefrontal Cortex; LMFG, Left Median Frontal Gyrus; RMFG, Left Median Frontal Gyrus; RSFG, Right Superior Frontal Gyrus; RSTG, Right Superior Temporal Gyrus*.

**Table 6:** Pearson correlations (r) between activations of ACC, SFG, and MFG.

**ROI**	**Time course**	**ACC [−400/−350 ms]**	**ACC [−350/−300 ms]**	**ACC [−300/−250 ms]**	**ACC [−250/−200 ms]**
RSFG	[−400/−350 ms]	0.609^*^	*ns*	*ns*	*Ns*
	[−350/−300 ms]	*ns*	0.724^**^	0.559^*^	*ns*
LSFG	[−350/−300 ms]	0.537^*^	*ns*	*ns*	0.731^**^
RMFG	[−400/−350 ms]	0.708^**^	0.675^**^	*ns*	0.658^*^
	[−350/−300 ms]	0.573^**^	*ns*	0.572^*^	*ns*
	[−250/−200 ms]	0.565^*^	0.688^**^	0.551^*^	0.627^*^
LMFG	[200/250 ms]	*ns*	0.645^*^	0.647^*^	0.569^*^

*ACC, Anterior Cingulate Cortex; LMFG, Left Median Frontal Gyrus; RMFG, Left Median Frontal Gyrus; RSFG, Right Superior Frontal Gyrus; LSFG, Left superior Frontal Gyrus*.

* *p < 0.05*,

** *p < 0.01, ns, non-significant*.

#### Comparisons of common regions between young adults and high-functioning older adults

Second, in line with Hinault et al.'s results (Braver et al., 2009; Hinault et al., 2017), we expected to observe age-related changes in ACC and LIFJ activations. For each ROI, we conducted a 2 (Group: Young adults, older adults) × 2 (Strategy on the previous problems: Poorer, better) × 19 (Times) mixed design ANOVA on current poorer strategy problems. Analyses showed a significant Group x Strategy on the previous Problems × Times interaction for the ACC, [*F*_(18.468)_ = 1.929*, Mse* = 2.509, η^2^_*p*_ = 0.069*, p* = 0.012], but not for the LIFJ [*F*_(18.468)_ = 0.697*, Mse* = 2.191, η^2^_*p*_ = 0.026*, p* = 0.815]. In contrast to young adults, who showed engagement of ACC during the processing of poorer strategy problems following better strategy problems, analyses revealed ACC activations in older adults when the previous problems were solved with the poorer strategy, before the onset of the current poorer strategy problems. Results revealed that larger ACC activations following previous better strategy problems than poorer strategy problems occurred earlier and longer in older adults than in young adults.

## Discussion

In this study, we benefited from the excellent temporal and spatial resolutions of MEG to investigate age-related changes in the neural bases of sequential strategy interference effects. MEG data revealed that older adults, in addition to activating similar brain regions than young adults (i.e., ACC and LIFJ), recruited additional brain areas in frontal, parietal, and temporal cortex. Also, older adults recruited these brain areas with specific temporal dynamics. These age-related differences in the spatial-temporal dynamics of brain activations were found while young adults and older adults showed similar behavioral sequential modulations of strategy interference effects. These results have important implications for our understanding how young and older participants execute cognitive strategies, in general, and of sequential strategy interference effects during aging in particular.

In the majority of previous studies that showed additional brain activations in older adults associated with similar performance to young adults, the type of strategies that young and older adults used was not assessed or controlled (e.g., Grady et al., 1994; Reuter-Lorenz et al., 2000; Cabeza, 2002; Cabeza et al., 2002; Logan et al., 2002; Madden, 2007; Reuter-Lorenz and Park, 2014; Angel et al., 2015). It was therefore not possible to determine whether age-related differences in brain activations corresponded to differences in strategy repertoire and/or strategy execution. Indeed, differences in strategy repertoire (i.e., differences between young and older in which strategies were used to accomplish cognitive tasks or in how many strategies were used across all items) may yield differences in brain activations (e.g., El Yagoubi et al., 2005; Gandini et al., 2008; see Lemaire, 2016, for an overview). Conversely, differences in how young and older adults execute the same strategies may also result in differences in brain activations (i.e., with more brain areas activated to execute the same strategies in older adults than in young adults; e.g., Logan and Buckner, 2001; Gandini et al., 2008). Here, we controlled which strategy young and older participants used on each problem, and ensured that the observed brain activations could not result from differences in strategy repertoire. Therefore, the present results provide strong evidence that recruitments of additional brain areas by older adults helped them to execute strategies as efficiently as young adults.

In older adults, we observed differences in brain activations during the processing of the current poorer strategy problems, as a function of the strategy used on the immediately preceding better or poorer strategy problems. Following the execution of a poorer strategy, older adults recruited the same brain areas as young adults (i.e., LIFJ) in a similar way, but they also recruited additional areas in frontal and temporal brain regions. More specifically, older adults engaged contralateral brain regions such as the right inferior frontal junction (i.e., RIFJ), previously associated with inhibitory processes (e.g., Hampshire et al., 2010). This finding is consistent with previous fMRI studies that showed bilateral frontal activations in older adults relative to young adults (e.g., Grady et al., 1994; Reuter-Lorenz et al., 2000; Cabeza, 2002; Cabeza et al., 2002; Gandini et al., 2008; Reuter-Lorenz and Park, 2014; Angel et al., 2015; see Cabeza and Dennis, 2012; for a review). According to the HAROLD model (Cabeza, 2002), older adults recruit bilateral areas to compensate for age-related decline in cognitive abilities, especially regarding inhibitory mechanisms, and to maintain similar performance than young adults (e.g., Nielson et al., 2002).

In the present experiment, in contrast to young adults, who showed ACC activations when no preparation was engaged (i.e., after the execution of a better strategy), ACC activations were found in older adults after execution of a poorer strategy. Furthermore, ACC activations correlated positively with activations in frontal regions, like the median and superior frontal gyri. Previous fMRI studies revealed that, in addition to the detection of conflict, the ACC was also involved in conflict resolution, to increase the level of control (e.g., Carter et al., 1998; Van Veen et al., 2001; Nee et al., 2007; Shallice et al., 2008). The present results, together with correlations between ACC and frontal areas, are in line with this interpretation. Therefore, it appears that, following the resolution of a conflict, older adults engaged the ACC in a proactive way to ensure a more efficient interference processing on the next problems.

The present results also revealed activations in the right and left superior frontal gyri, the right median frontal gyrus, and in the temporal gyrus. These brain regions were previously associated with working memory maintenance (i.e., Cornette et al., 2001; Picchioni et al., 2007; Verbruggen and Logan, 2008; Rose et al., 2015). These results suggest that older adults recruited working-memory areas to efficiently modulate sequential strategy interference effects. These working memory processes would enable older adults to maintain relevant information activated in working memory (e.g., which strategies to execute), to focus their attention on this information more efficiently, and to be less influenced by irrelevant information (e.g., activation of the better strategy triggered by unit digits in each operand). These additional activations enabled older adults to prepare themselves from one problem to the next to efficiently execute the cued strategy on the next problem (i.e., proactive control).

After the execution of the better strategy, older adults also showed additional brain activations relative to young adults. Older adults showed activations in the right frontal median gyrus and in the left orbitofrontal region, in the prefrontal cortex, previously associated with conflict detection and decision making (e.g., Bechara et al., 2000; Verbruggen and Logan, 2009). Moreover, older adults mobilized the left inferior parietal lobule. Parietal regions have been found to be involved in a variety of arithmetic problem solving task. Alternatively, the implementation of executive control such as conflict detection and decision making could involve other left frontal areas (i.e., ACC and LOFC, respectively). These additional recruitments suggest that older adults rely more strongly on both general (e.g., executive control mechanisms) and specific arithmetic processes to execute the cued strategy.

Similarly to young adults (e.g., Carter et al., 2000; Brass and Von Cramon, 2004; Kerns et al., 2004; Brass et al., 2005; Montojo and Courtney, 2008; Braver et al., 2009; Hinault et al., 2017), older adults showed activations of the ACC and LIFJ during sequential modulations of conflict processing. However, older adults also showed the engagement of additional brain areas in frontal, temporal, and parietal regions. These fronto-temporal and fronto-parietal activations are line with the executive network described in the Scaffolding Theory of Cognitive Aging (Huang et al., 1999; Daselaar et al., 2006; Reuter-Lorenz and Park, 2014; Angel et al., 2015). According to this model, to prevent aging effects, some older adults recruit additional brain areas to accomplish cognitive tasks as efficiently than young adults. These results suggest that older adults need additional processes to execute the cued strategy when a conflict is detected, with no possible anticipation.

Older adults also showed distinct temporal dynamics of activations relative to young adults. In contrast to young adults, after the execution of the poorer strategy, older adults recruited frontal regions (i.e., prefrontal cortex, superior and median frontal gyri) both before and during the processing of the current problems (i.e., from −400 to −200 ms before the second problems and from 500 to 700 ms after the onset of the second problems). This suggests that older adults needed to engage the same brain network in two distinct processing steps to efficiently execute the cued strategy on the second problems. Indeed, previous works suggested that the processing of the operands increases the activation of the better strategy (Hinault et al., 2014), which would require additional engagements of the control network in older adults. Furthermore, following the execution of the better strategy, older adults also showed distinct temporal dynamics of activations relative to young adults. Although older adults showed, like young adults, two temporal windows of activations, these brain activations were found earlier in older adults (i.e., between 100 and 1,250 ms after the onset of the second problems in older adults vs. between 150 and 1,500 ms in young adults). It is possible that earlier modulations enabled “high-control” older adults to maintain similar behavioral performance to young adults during strategy sequential interference effects.

Theoretically, the present findings have important implications for our understanding of the spatial-temporal dynamics of additional brain areas recruited by older adults with high performance. Recent theories of cognitive aging may be helpful in explaining these results. The STAC-r model (i.e., Scaffolding Theory of Cognitive Aging; Reuter-Lorenz and Park, 2014; GOLDEN, Growth of Lifelong Differences Explains Normal Aging; Fabiani, 2012) was proposed to integrate the current theories of compensatory mechanisms. This model assumes that aging leads to changes at the neural level, which are associated with a decline in cognitive functioning. To maintain high level of cognitive functioning, compensatory mechanisms can be implemented, with changes in brain networks underlying task performance.

The present study, together with recent works (e.g., Deary et al., 2006; Gandini et al., 2008; Davis et al., 2009; Collette and Salmon, 2014; Reuter-Lorenz and Park, 2014; Angel et al., 2015; Hinault et al., 2016a, 2017) suggest the involvement of cognitive control processes in the maintenance of cognitive performance with age. Indeed, “high-control” older adults showed activations in additional brain areas in frontal, temporal, and parietal regions previously associated with cognitive control mechanisms (e.g., Cornette et al., 2001; Daselaar et al., 2006; Picchioni et al., 2007; Verbruggen and Logan, 2008; Huang et al., 2012; Rose et al., 2015). However, current models of neurocognitive aging are mainly based on fMRI and EEG data and do not document the spatial-temporal dynamics of additional neural recruitments in older adults. Models should take into account the present MEG results, as they revealed that age-related changes of brain functioning in space, with the activation of additional brain areas relative to young adults, also occur in time, with the engagement of this extended control network both (a) earlier to prepare interference processing, and (b) later to compensate for the reduced efficiency of cognitive control processes. All in all, these results shed new lights on the preservation of behavioral performance in high-performing older adults.

## Author contributions

AR collected, analyzed the data, and contributed to the write-up of the manuscript. TH and PL contributed to the manuscript write-up and study design as well as to statistical analyses. J-MB contributed to study design and statistical analyses. All the authors approved the final manuscript.

### Conflict of interest statement

The authors declare that the research was conducted in the absence of any commercial or financial relationships that could be construed as a potential conflict of interest.
